# *Anopheles* vector distribution and malaria transmission dynamics in Gbêkê region, central Côte d’Ivoire

**DOI:** 10.1186/s12936-023-04623-1

**Published:** 2023-06-22

**Authors:** Alphonsine A. Koffi, Soromane Camara, Ludovic P. Ahoua Alou, Welbeck A. Oumbouke, Rosine Z. Wolie, Innocent Z. Tia, Eleanore D. Sternberg, Florent H. A. Yapo, Fernand M. Koffi, Serge B. Assi, Jackie Cook, Matthew B. Thomas, Raphael N’Guessan

**Affiliations:** 1 Institut Pierre Richet (IPR)/Institut National de Santé Publique (INSP), Bouaké, Côte d’Ivoire; 2 Vector Control Product Evaluation Centre (VCPEC), Institut Pierre Richet (VCPEC-IPR)/INSP, Bouaké, Côte d’Ivoire; 3 Innovative Vector Control Consortium, IVCC, Liverpool, UK; 4 Unité de Recherche et de Pédagogie de Génétique, Université Félix Houphouët-Boigny, UFR Biosciences, Abidjan, Côte d’Ivoire; 5 Centre d’Entomologie Médicale et Vétérinaire, Université Allassane Ouattara, Bouaké, Côte d’Ivoire; 6 Tropical Health LLP, London, UK; 7 MRC International Statistics and Epidemiology Group, London School of Hygiene and Tropical Medicine, London, UK; 8 Department of Entomology & Nematology, The University of Florida, Gainesville, FL USA; 9 Department of Disease Control, London School of Hygiene and Tropical Medicine, London, UK

**Keywords:** *Anopheles* vector, Malaria transmission, Insecticide resistance, Côte d’Ivoire

## Abstract

**Background:**

A better understanding of vector distribution and malaria transmission dynamics at a local scale is essential for implementing and evaluating effectiveness of vector control strategies. Through the data gathered in the framework of a cluster randomized controlled trial (CRT) evaluating the In2Care (Wageningen, Netherlands) Eave Tubes strategy, the distribution of the *Anopheles* vector, their biting behaviour and malaria transmission dynamics were investigated in Gbêkê region, central Côte d’Ivoire.

**Methods:**

From May 2017 to April 2019, adult mosquitoes were collected monthly using human landing catches (HLC) in twenty villages in Gbêkê region. Mosquito species wereidentified morphologically. Monthly entomological inoculation rates (EIR) were estimated by combining the HLC data with mosquito sporozoite infection rates measured in a subset of *Anopheles* vectors using PCR. Finally, biting rate and EIR fluctuations were fit to local rainfall data to investigate the seasonal determinants of mosquito abundance and malaria transmission in this region.

**Results:**

Overall, *Anopheles gambiae*, *Anopheles funestus*, and *Anopheles nili* were the three vector complexes found infected in the Gbêkê region, but there was a variation in *Anopheles* vector composition between villages. *Anopheles gambiae* was the predominant malaria vector responsible for 84.8% of *Plasmodium* parasite transmission in the area. An unprotected individual living in Gbêkê region received an average of 260 [222–298], 43.5 [35.8–51.29] and 3.02 [1.96–4] infected bites per year from *An. gambiae, An. funestus* and *An. nili*, respectively. Vector abundance and malaria transmission dynamics varied significantly between seasons and the highest biting rate and EIRs occurred in the months of heavy rainfall. However, mosquitoes infected with malaria parasites remained present in the dry season, despite the low density of mosquito populations.

**Conclusion:**

These results demonstrate that the intensity of malaria transmission is extremely high in Gbêkê region, especially during the rainy season. The study highlights the risk factors of transmission that could negatively impact current interventions that target indoor control, as well as the urgent need for additional vector control tools to target the population of malaria vectors in Gbêkê region and reduce the burden of the disease.

## Background

Malaria is still a major public health problem in sub-Saharan Africa despite improvements in the diagnosisof the pathogens and large-scale deployment of vector control tools, such as long-lasting insecticidal nets (LLINs) and indoor residual spraying (IRS). According to the World Malaria Report 2021, a slight upward trend in malaria incidence was observed in 2020, after stagnation, between 2015 and 2019 [1]. A number of factors may contribute to this, including the growing problem of insecticide-resistant mosquitoes [2, 3], outdoor malaria transmission [4, 5], residual transmission [6, 7], gaps in control management [8] and disruption to services during the COVID-19 pandemic [1].

Today, the greatest burden of malaria occurs across in the World Health Organization (WHO) African region, with an estimated 228 million malaria cases and 602,000 malaria deaths [1]. Malaria in sub-Saharan Africa is transmitted by a range of *Anopheles* mosquitoes [9, 10] and transmission dynamics can be highly heterogenous [11, 12]. Although the whole sub-Saharan region is exposed to malaria transmission, the risk of infection and disease varies greatly across the continent and even within small geographical areas [11, 13]. This high heterogeneity, influenced by ecological factors, such as climate, physical geography, land use, human behaviour and other social factors [14–16], needs to be considered when planning and implementing vector control strategies.

In Côte d’Ivoire, malaria transmission is perennial, albeit with a sharp increase during the wet season [17, 18]. The *Plasmodium* species responsible for human malaria are mainly transmitted by the primary vectors *Anopheles gambiae *sensu stricto and *Anopheles coluzzii* [19]. *Anopheles funestus *sensu lato (*s.l*.) and *Anopheles nili s.l.* are secondary vectors [17, 20]. In some localities of western Côte d’Ivoire, these secondary vectors have played a significant role in malaria transmission largely due to their predominantly anthropophilic and endophilic tendencies [20]. Malaria incidence was estimated at more than 287 cases per thousand and 15,913 deaths in 2020 [1]. Recently in Gbêkê region, central Côte d’Ivoire the incidence of malaria infection has been estimated at 2.29 per child-year [21].

Vector control by the national malaria control programme (NMCP) is based on sustaining high LLIN access and use, via universal coverage campaigns supplemented with continuous distribution from antenatal care campaigns and the expanded programme for immunization; targeted IRS in high transmission areas since 2020 and treatment. Since 2010, the NMCP, with the support of the Global Fund to Fight AIDS, Tuberculosis and Malaria (Global Fund), started scaling up mass distribution of LLINs to achieved universal coverage. Unfortunately, the large scale use of pyrethroid insecticides in public health as well as in agriculture has resulted in mosquitoes building up high resistance to the insecticide [22–24], making the pyrethroid-treated nets less effective [25–27]. In the face of increasing pyrethroid resistance, many African countries NMCPs are challenged with finding new ways to prevent malaria transmission. Urgent action is required to slow or prevent the development and further spread of insecticide resistance, including the use of two or more compounds of different insecticide classes to make a single product or development and evaluation of new interventions strategies that aim to maintain effective vector control [28].

In the Gbêkê region, central Côte d’Ivoire the In2Care (Wageningen, Netherlands) Eave Tubes, a new tool for the targeted delivery of insecticides against mosquitoes, attempting to enter houses through the eaves have been evaluated in a large-scale cluster randomized trial (CRT) between May 2017 and April 2019 [21]. The project, which was conducted in 40 villages, was designed to test whether modification of houses through the addition of window screening and eave tubes, provides additional protection against malaria in areas with intense pyrethroid-resistance above and beyond universal coverage of pyrethroid-only LLINs [29]. The epidemiological results of the study published recently, showed an impressive drop of 38% in malaria case incidence in children living in clusters with intervention [21]. Through the data gathered within this trial in the 20 control villages, vector distribution, their behaviour and malaria transmission dynamic were updated in Gbêkê region, under natural conditions with universal coverage of pyrethroid-only bed nets.

## Methods

### Study area and trial design

The study was carried out in the Gbêkê region in central Côte d’Ivoire. This region is characterized by wet savannah with a single annual rainy season (April to November) followed by a long dry season (December to March). There was an average annual rainfall of 1223 mm and an average temperature of 26.3  C during the study period. It is highly malaria endemic area with year-round transmission, and malaria cases are almost entirely attributable to *Plasmodium falciparum* [30, 31]. Members of the *An. gambiae* complex (*An. coluzzii* and *An. gambiae s.s.*) are the main vectors [30]. Members of the *An. funestus* were also present as secondary vectors [21]. The local malaria vector populations are highly resistant to almost all classes of insecticides used for vector control [22, 23, 32].

The eave tube study design was a two-armed, cluster-randomized controlled trial with 20 villages (clusters) per arm. Villages in the control arm received universal coverage of LLINs, while the villages in the intervention arm received universal coverage of LLINs plus the screening plus eave tube (SET) intervention free of charge [29]. In the framework of this study, only the data from control villages were analysed to show the natural transmission dynamics.

### Mosquito sampling

**Fig. 1:**
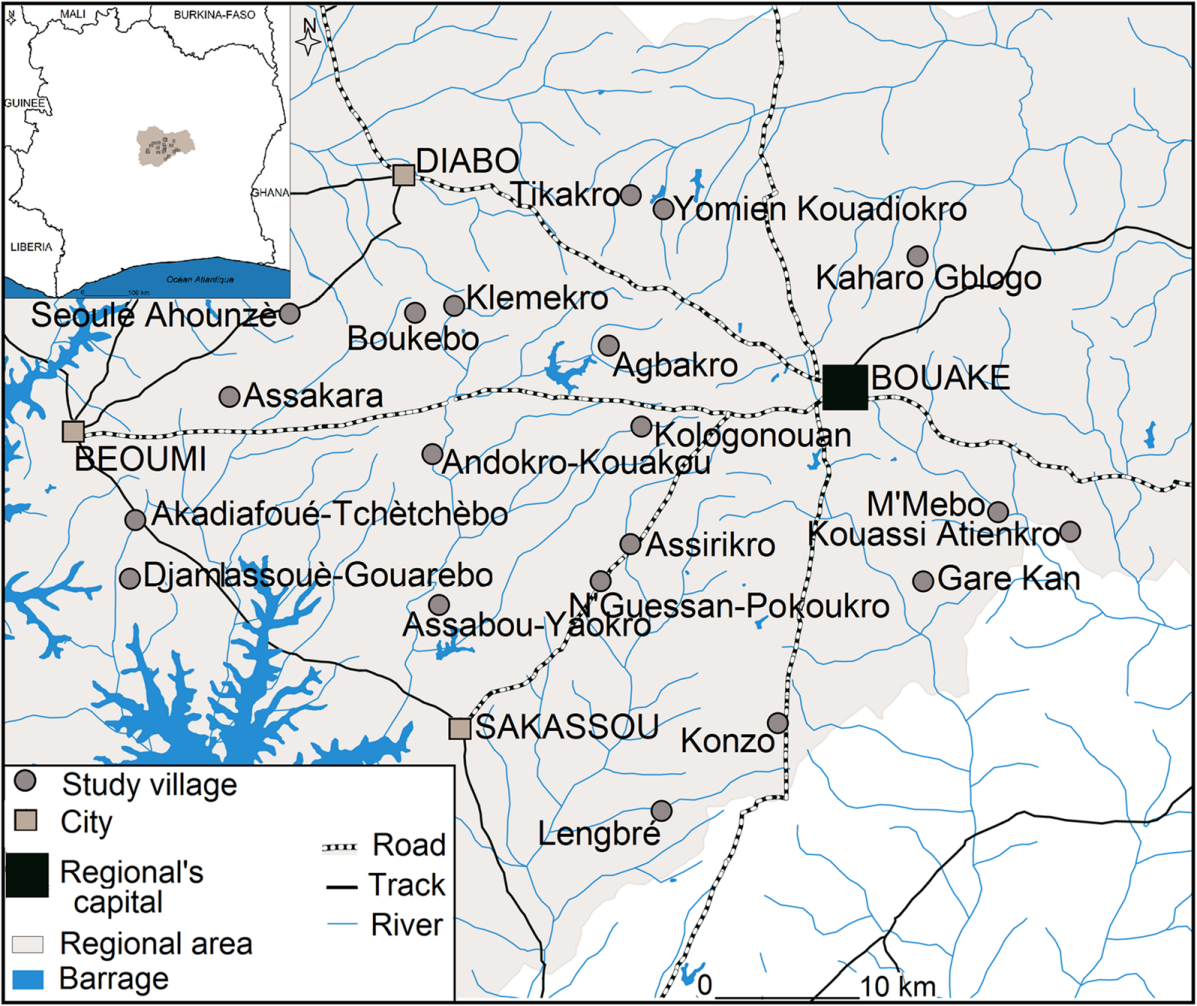
A map of study area, showing sampling villages

Each month between May 2017 and April 2019, mosquitoes were sampled using human landing catches (HLC) both indoors and outdoors for one night at four randomly selected houses in the 20 study villages (Fig. 1) to estimate the variation in their abundance over time. Collections began at 06:00 pm with one person sitting inside of the house in the living room area and one sitting outside of the house. Every hour, each team were supervised by research technicians to ensure that they were awake and working according to protocol. Every two hours, the two capturers were rotated between indoor and outdoor collection sites to minimize the bias due to their attractiveness. At 01:00 am, a second capturer team took over and continued the capture until 08:00 am. Mosquitoes collected were brought back to the laboratory, and were identified using a species key based on morphological traits[33]. Mosquitoes were stored individually in tubes with silica gel and kept at − 20 °C pending further laboratory processing. Non-*Anopheles* species were discarded after recording numbers caught.

Monthly rainfall and temperature data during the study period was obtained from the National Weather Service of Bouake airport. The data consist of monthly mean of the daily rainfall and temperature in the region.

### Determining *Plasmodium* spp infection in female mosquitoes

A random subset of 59,901 captured *An. gambiae s.l.* and 10,059 *An. funestus* and 1,991 *An. nili* females were dissected to determine parity. Mosquito DNA was extracted from the head and thorax of each specimen in a random sample of up to 60 parous females per village per monthly survey using cetyl trimethyl ammonium bromide (CTAB) 2% method [34]. Quantitative Polymerase Chain Reaction (qPCR) was used to assess sporozoite prevalence as described by Mangold et al*.* [35].

### Data analysis

Human landing catch captures were done monthly and then the data were pooled every two months for analysis. The human biting rate (HBR, the number of Anopheles vectors collected per person per night), the sporozoite rate (SR, the number of vectors positive for sporozoites over the number of vectors tested) and the entomological inoculation rate (EIR, is the number of infective bites per person per night). EIR was calculated by multiplying the HBR by the SR as described by Sternberg et al*.* All non-parous mosquitoes were considered negative for the calculation of sporozoite rates and EIRs.

A separate rate was determinedfor HBR, SR and EIR for each species.

Statistical analysis for the comparison of HBR, SRs and EIR between species, seasons (rainy/dry), year and collection positions (indoor/outdoor) were performed using R software version 4.1.2, and figures with GraphPad Prism 7 software.

To assess the difference in HBR and EIR, a generalized linear mixed model (GLMM) fitting a negative binomial distribution was applied using the *lme4* package. SRs were compared using a binomial mixed effect model (function “glmer” from the package *lme4*) [36]. The fixed variables were the *Anopheles* species, collection position (indoor/outdoor), season (rainy/dry) and year. The villages and month of collection were considered as a random intercept to adjust for sampling variations across villages and years.

## Results

### Species composition and vector distribution

A total of 157,645 mosquitoes belonging to four genera were collected over 4,880 sampling person-nights using HLC methods (Table 1). Of these, 71,207 (45.2%) were collected indoors and 86,438 (54.8%) outdoors. Mosquitoes collected included *Anopheles, Aedes, Culex* and *Mansonia* species (Table 1). Of the Anophelines collected, 94.5% (128,632/136,049) were malaria vectors comprised of *An. gambiae*, *An. funestus* and *An. nili.*

**Table 1:** Diversity and abundance of mosquito species from 20 villages in Gbêkê region from May 2017 to April 2019

Species	Total mosquito collected indoor	Total mosquito collected outdoor	Total collected	%
*An. gambiae* s.l	50892	57869	108761	69.0
*An. funestus* s.l	8127	7290	15417	9.8
*An. nili* s.l	1374	3080	4454	2.8
Other *Anopheles* spp.	2893	4524	7417	4.7
*Aedes sp.*	119	294	413	0.3
*Culex sp.*	2356	4417	6773	4.3
*Mansonia sp.*	5446	8964	14410	9.1
Total	71207	86438	157645	100

Overall, *An. gambiae s.l.* was the most common malaria vector, and accounted for more than 84.5% (108,761/128,632) of all malaria vectors. The other vectors were *An. funestus* (12.0% (15,417/128,632)) and *An. nili* 3.5% (4,454/128,632) (Table 2).

**Table 2:** Entomological outcomes by season and collection location for *Anopheles* vector from may 2017 to April 2019

Outcomes	*Anopheles gambiae s.l*	*Anopheles funestus* group	*Anopheles nili* complex	Overall malaria vector
Total collection nights	4880	4880	4880	4880
Total mosquitoes collected (%)	108761 (84.5)	15417 (12.0)	4454 (3.5)	128,632
Total collected indoor (%)	50892 (46.8)	8127 (52.7)	1374 (30.8)	60,393 (46.9)
Total collected outdoor (%)	57869 (53.2)	7290 (47.3)	3080 (69.1)	68,239 (53.1)
Mean mosquito density [95% CI]	18.0 [11.0–26.8]	2.5 [0.8–4.1]	0.5 [-0.1–1.1]	21.7 [14.6–28.7]
Indoor mosquito density [95% CI]	17.2 [11.8–22.6]	2.7 [1.4–3.9]	0.3 [0.0–0.6]	20.2 [18.2–22.2]
Outdoor mosquito density [95% CI]	20.1 [13.8–26.3]	2.5 [1.4–3.5]	0.7 [0.1–1.3]	23.2 [21.0–25.5]
Mean density in rainy season [95% CI]	24.9 [22.5–27.3]	3.6 [3.1–4.2]	0.7 [0.5–1.0]	32.4 [29.0–35.8]
Mean density in dry season [95% CI]	7.0 [5.7–8.3]	0.15 [0.10–0.20]	0	7.2 [5.8–8.4]
Total dissected	59901	10059	1991	71,951
Parity rate [95% CI], Total parous	83.3 [83.0–83.6], 49923	88.9 [88.2–89.5], 8943	97.2 [96.4–97.9],1937	84.9 [84.6–85.1], 60,803
Total tested for sporozoites	9856	4145	489	14,490
Number of sporozoite positive	453	239	11	703
Overall SR % [95% CI]	4.6 [4.2–5.0]	5.8 [5.0–6.5]	2.2 [1.0–3.6]	4.8 [4.5–5.2]
% SR indoor [95% CI]	5.1 [4.4–5.7]	5.8 [4.8–6.9]	1.5 [0–3.2]	5.1 [4.2–6.0]
% SR outdoor [95% CI]	4.1 [3.6–4.7]	5.7 [4.7–6.6]	2.8 [1.0–4.7]	4.4 [3.7–5.2]
% SR rainy season [95% CI]	5.1 [4.6–5.6]	5.8 [5.0–6.5]	2.2 [0.9–3.6]	5.1 [4.2–6.0]
% SR dry season [95% CI]	2.4 [1.7–3.1]	5.9 [0.3–11.5]	0	2.8 [2.0–3.7]
Mean monthly EIR (weighted)	21.7 [10.4–33.1]	3.6 [1.3–5.9]	0.2 [0–0.5]	31.0 [26.8–35.1]
EIR rainy season [95% CI]	30.6 [27.3–33.9]	5.4 [4.7–6.1]	0.4 [0.2–0.5]	38.2 [35.0–41.4]
EIR dry season [95% CI]	3.9 [2.4–5.4]	0.1 [0.0–0.2]	0	4.3 [2.5–6.0]
Estimate annual EIR (weighted)	260.0 [222.0–298.0]	43.5 [35.8–51.3]	3.0 [2.0–4.0]	321.0 [278.0–365.0]
% EIR contribution (weighted)	84.8%	14.9	0.98	

SR: Sporozoite rate, EIR: mean entomological inoculation rate, [95% CI]: 95% Confidence interval

**Fig. 2:**
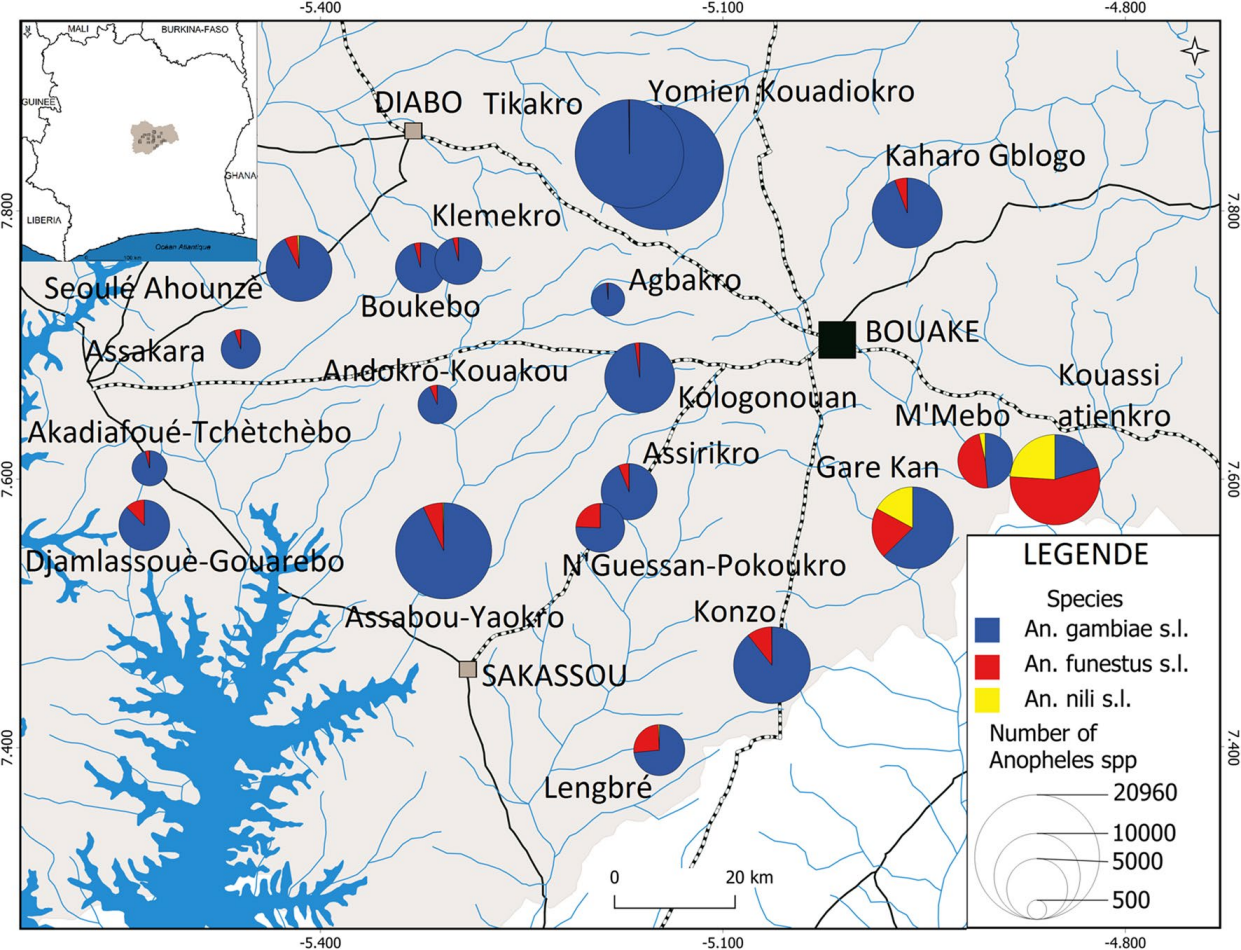
A map of *Anopheles* densities and composition in the study area

The relative abundance and species composition of the malaria vectors varied from one village to another (Table 3; Fig. 2). In most of the study villages *An. gambiae* predominated throughout the year with more than 70% of the catch, followed by *An. funestus* and *An. nili*. Exceptions were seen in 3 villages, *An. funestus* and *An. nili* were main malaria vectors found in Kouassi Atienkro with 55.5% and 24.0% of the catch, respectively. In M’Mebo *An. gambiae* (48.6% of catch) and *An. funestus* (47.9% of the catch) were equally present. In Gare Kan village, *An. gambiae* was the most abundantly represented with 62.7% of malaria vector collected, but *An. funestus* (20.2%) and *An. nili* (17.2%) were also found at a comparable rate (Table 3; Fig. 2).

**Table 3:** Distribution of malaria vector and *Plasmodium* infection rate according to sampling villages in Gbèkè region

Sampling villages	Malaria vector distribution Total (%)			Total	Sporozoite rate		
	*An. gambiae sl*	*An. funestus group*	*An. nili* complex		Total Tested	N. infected	SR [95% CI]
Agbakro	1472 (98.6)	19 (1.3)	2 (0.1)	1493	417	26	6.2 [9.91–8.55]
Andokro Kouakou	1904 (93.6)	128 (6.3)	2(0.1)	2034	559	58	10.4 [7.84–12.90]
Boukebo	3266 (95.8)	141 (4.1)	1 (0.03)	3408	550	14	2.5 [1.23–3.85]
Klemekro	2880 (96.1)	113 (3.8)	3 (0.1)	2996	483	22	4.5 [2.69–6.41]
Kologonouan	6501 (97.8)	139 (2.1)	8 (0.1)	6648	713	46	6.4 [4.65–8.25]
Tikakro	15897 (99.8)	36 (0.2)	2 (0.01)	15935	627	23	3.7 [2.19–5.13]
Yomien Kouadiokro	20925 (99.8)	33 (0.2)	2 (0.01)	20960	672	16	2.4 [1.23–3.53]
Kaharo gblogo	6261 (94.2)	384 (5.8)	2 (0.03)	6647	752	42	5.6 [3.94–7.22]
Kouassi Atienkro	2246 (20.5	6072 (55.5)	2623 (24.0)	10941	1129	42	3.7 [2.62–8.82]
M'MEBO	1971 (48.6)	1943 (47.9)	145 (3.6)	4059	860	57	6.6 [4.96–8.28]
Gare Kan	5618 (62.7)	1812 (20.2)	1535 (17.1)	8965	1229	57	4.6 [3.46–5.80]
Konzo	7099 (89.3)	844 (10.6)	7 (0.1)	7950	882	53	6.0 [4.43–7.57]
Lengbre	2587 (73.5)	909 (25.8)	24 (0.7)	3520	787	45	5.7 [4.09–7.33]
Akadiafoué-tchètchèbo	1606 (95.9)	67 (4.0)	2 (0.1)	1675	469	31	6.6 [4.36–8.66]
Assakara	1976 (94.7)	108 (5.2)	3 (0.1)	2087	390	14	3.6 [1.72–5.42]
Djamlassoué-Gouarebo	3067 (87.8)	426 (12.2)	0 (0)	3493	734	33	4.5 [2.99–5.99]
Seoule Ahounzè	5448 (92.9)	370 (6.3)	49 (0.8)	5867	762	27	3.5 [2.23–4.85]
Assirikro	3976 (93.8)	260 (6.1)	3 (0.1)	4239	722	41	5.7 [3.98–7.36]
Assabou yaokro	11617 (93.0)	829 (6.6)	39 (0.3)	12485	997	28	2.8 [1.78–3.89]
N'Guessan Pokoukro	2444 (75.7)	784 (24.3)	2 (0.1)	3230	862	37	4.3 [2.94–5.64]

N. infected: Number infected; SR: Sporozoite rate; [95% CI]: 95% Confidence interval

### Dynamics of malaria transmission

#### Seasonal abundance and biting patterns of Anopheles mosquitoes

**Fig. 3:**
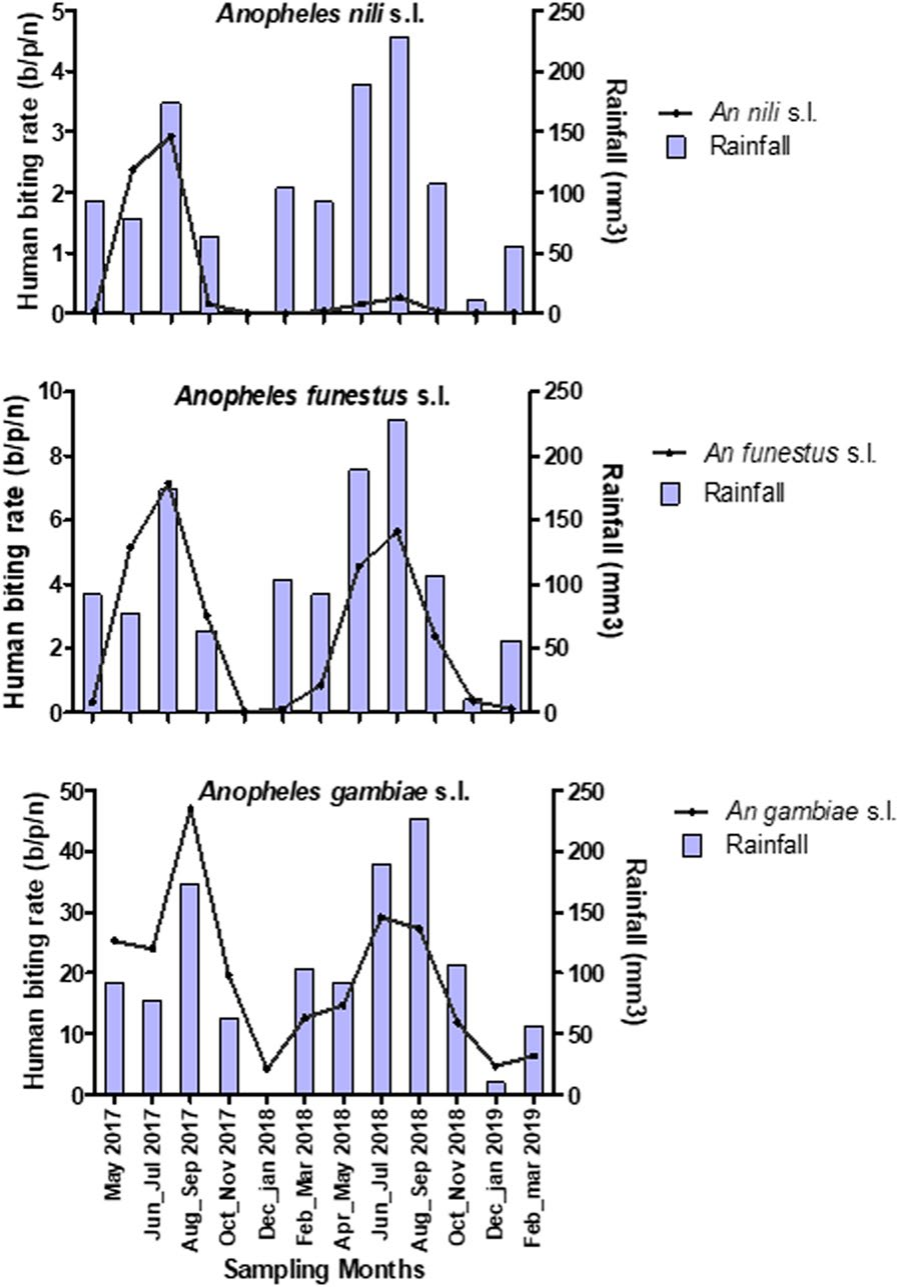
Monthly abundances of human-biting Anopheles species in Gbêkê region

The monthly abundance of human-biting *Anopheles* species during the study period are shown in Fig. 3. The mean *Anopheles* caught per human, per night were: *An. gambiae* = 18.0 [95% CI 11.0–26.8], *An. funestus* = 2.5 [95% CI 0.8–4.1] and *An. nili* = 0.5 [95% CI − 0.1–1.5] (Table 2). Significantly greater numbers of *An. gambiae* were collected across the study area compared to the *An. funestus* group (RR [95%] = 12.6 [12.0–13.1] p < 0.001) and the *An. nili* group (RR [95%] = 56.4 [53.0–60.1], p < 0.001) (Table 2). Overall, *An. gambiae* and *An. nili* biting rates were significantly higher outdoors compared to indoors (OR [95% CI] = 1.2 [1.16–1.23], p < 0.001) suggesting an exophilic tendency for these species in this study. In contrast, the highest biting rates in *An. funestus* group were recorded indoors compared to outdoors (OR [95% CI] = 0.9 [0.86–0.98], p = 0.014) confirming that *An. funestus* tends to be endophagic (Table 2).

During the sampling period, more vectors (of the *An. gambiae*, *An. funestus* and *An. nili* group) were recorded during the rainy season (April to November) than in the dry season (December to March) (RR [95%] = 4.0 [2.2–7.3], p < 0.001), but vectors biting rates peaked in August and September corresponding to rainiest months (Fig. 3).

Mean density of *An. gambiae* per human per night was 7.0 [5.7–8.3] during the dry season, but increased three-fold (24.9 [22.5–27.3]; p = 0.010) in the rainy season. Overall, densities of *An. funestus* and *An. nili* were very low and were closely correlated with monthly rainfall patterns (Fig. 3). Both vectors were almost undetectable during the dry seasons (December to March) (Fig. 3).

**Fig. 4:**
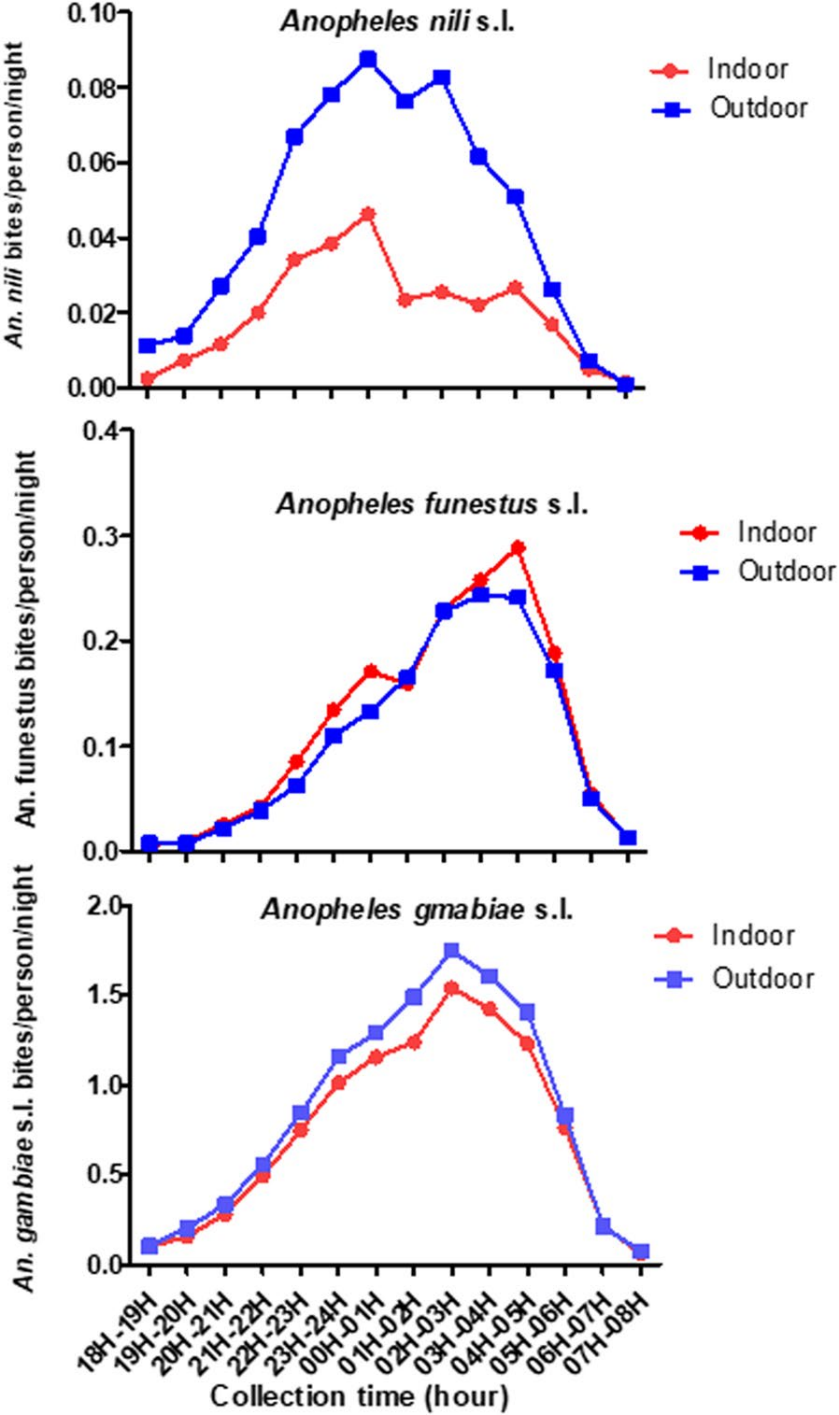
Hourly catches of Anopheles vector at different hours of the night in Gbêkê region

Abundance of malaria vectors varied from one year to the other (Table 4). Overall the mean density of *An. gambiae* per person per night decreased significantly over the two years of data collection, from 22.1 [18.3–25.9] in year 1 period to 15.7 [13.0–18.4] in the year 2 of the trial period (RR [95%] = 0.58 [0.55–0.60], p < 0.001). There was no difference in *An. funestus* biting rates between years (p = 0.051; Table 4). For *An. nili*, the highest densities were observed during the first year of data collection (RR [95%] = 16.3 [13.3–20]. *Anopheles gambiae*, *An. funestus* and *An. nili* were recorded as biting all night long. However, *An. gambiae* peak of biting time was recorded between 02:00 am and 03:00 am indoors and outdoors, while that of *An*. *funestus* was recorded one hour later (between 04:00 am and 05:00 am) (Fig. 4). *Anopheles funestus* was also recorded biting predominantly indoors during the night. *An. nili* showed earlier biting activity (beginning at 11:00 pm) than *An. gambiae* and *An. funestus*, with biting densities increased between 00:00 am and 01:00 am, and then decreased during the second part of the night (Fig. 4).

**Table 4:** Entomological outcomes for *Anopheles* vector according to data collection year

Outcomes	Year1 collected data (May 2017–March 2018)	Year 2 collected data (April 2018–March 2019)	Odds ratio	p value
*Anopheles gambiae* s.l				
Total collected (%)	81,480	27,281		
Mean mosquito density [95% CI]	22.1 [18.3–25.9]	15.7 [13.0–18.4]	0.58 [0.5–0.6]	< 0.0001
Total tested for sporozoites	5018	4838		
Number of sporozoite positive	186	267		
Sporozoites rate [95% CI]	3.7 [3.2–4.2]	5.5 [5.0–6.2]	0.7 [0.5–0.8]	< 0.0001
Entomological inoculation rate	248 [200–297]	271 [205–339]		
*Anopheles funestus* group				
Total collected	43,679	24,640		
Mean mosquito density	2.6 [1.8–3.4]	2.3 [1.7–2.9]	1.1 [1–1.1]	0.0510
Total tested for sporozoites	1810	2335		
Number of sporozoite positive	90	149		
Sporozoites rate [95% CI]	4.9 [4.0–6.0]	6.4 [5.4–7.4]	0.8 [0.6–1.0]	0.064
Entomological inoculation rate	43.2 [30.6–55.8]	43.9 [32.6–55.1]		
*Anopheles nili* complex				
Total collected	4311	143		
Mean mosquito density	0.9 [0.6–1.3]	0.1 [0.05–0.10]	16.3 [13.2–20.0]	< 0.0001
Total tested for sporozoites	379	110		
Number of sporozoite positive	7	4		
Sporozoites rate [95% CI]	1.8 [0.49–3.21]	3.6 [0.1–7.1]	0.4 [0.1–1.4]	0.153
Entomological inoculation rate	5.0 [3–7]	1.0 [0.3–1.5]		

[95% CI]: 95% Confidence interval

#### Parity and sporozoite infection rate

We dissected 71,951 Anopheles for determination of parous rate. Overall, *Anopheles* parous rate was 84.9%. Parous rate was 83.3%, 88.9% and 97.2% for *An. gambiae*, *An. funestus* and *An. nili*, respectively (Table 2).

A total of 14,490 *Anopheles* mosquitoes (*An. gambiae*, *An funestus* and *An nili*) were analysed to assess for the presence of *Plasmodium* spp*.*, with 703 found infected, giving an overall sporozoite rate of 4.8% [95% CI 4.5–5.2]. Infective *Anopheles* mosquitoes were found in all twenty study villages with infection rates ranging from 2.5% to 10.4% (Table 3). Most infections were with *Plasmodium falciparum* (94.6%), and the remaining (5.4%) were infections with *Plasmodium ovale* and *Plasmodium malariae*. The sporozoite rates did not differ significantly between malaria vectors collected indoors 5.1% [4.2–6.0] or outdoors 4.4% [3.7–5.2], (OR [95% CI] = 1.1 [1.0–1.3], p = 0.06). Overall sporozoite rate varied significantly among *Anopheles* spp. (p < 0.01) and fluctuated across the seasons with the highest rates observed in the rainy season (OR [95% CI] = 1.8 [1.4–2.4], p < 0.001, Table 2). The sporozoite rate recorded for *An. gambiae* during rainy season (5.1% [95% CI 4.6–5.6]) were significantly higher compared to dry season (2.4% [95% CI 1.7–3.1]); (OR [95% CI] = 0.4 [0.3–0.6], p < 0.0001). However, the sporozoite rate recorded for *An. funestus* in rainy season 5.8% [95% CI 5.0–6.5] and dry season 5.9% [95% CI 0.3–11.5] did not indicate seasonal variation (OR [95% CI] = 1.7 [1.0–3.2], p = 0.10). The *An. nili* appeared to contribute to transmission mainly in the rainy season (Table 2).

When considering the collection years, the sporozoite rate of *An. gambiae*, recorded in year 1 (3.7% [95% CI 3.2–4.2]) was significantly lower than that of the year 2 (5.5% [95% CI 5.0–6.2]) (OR [95% CI] = 0.7 [0.5–0.8], p < 0.0001). For *An. funestus* and in *An. nili* no significant difference of sporozoite rate wasfound between the two years (p > 0.05; Table 4).

### Entomological inoculation rate (EIR)

**Fig. 5:**
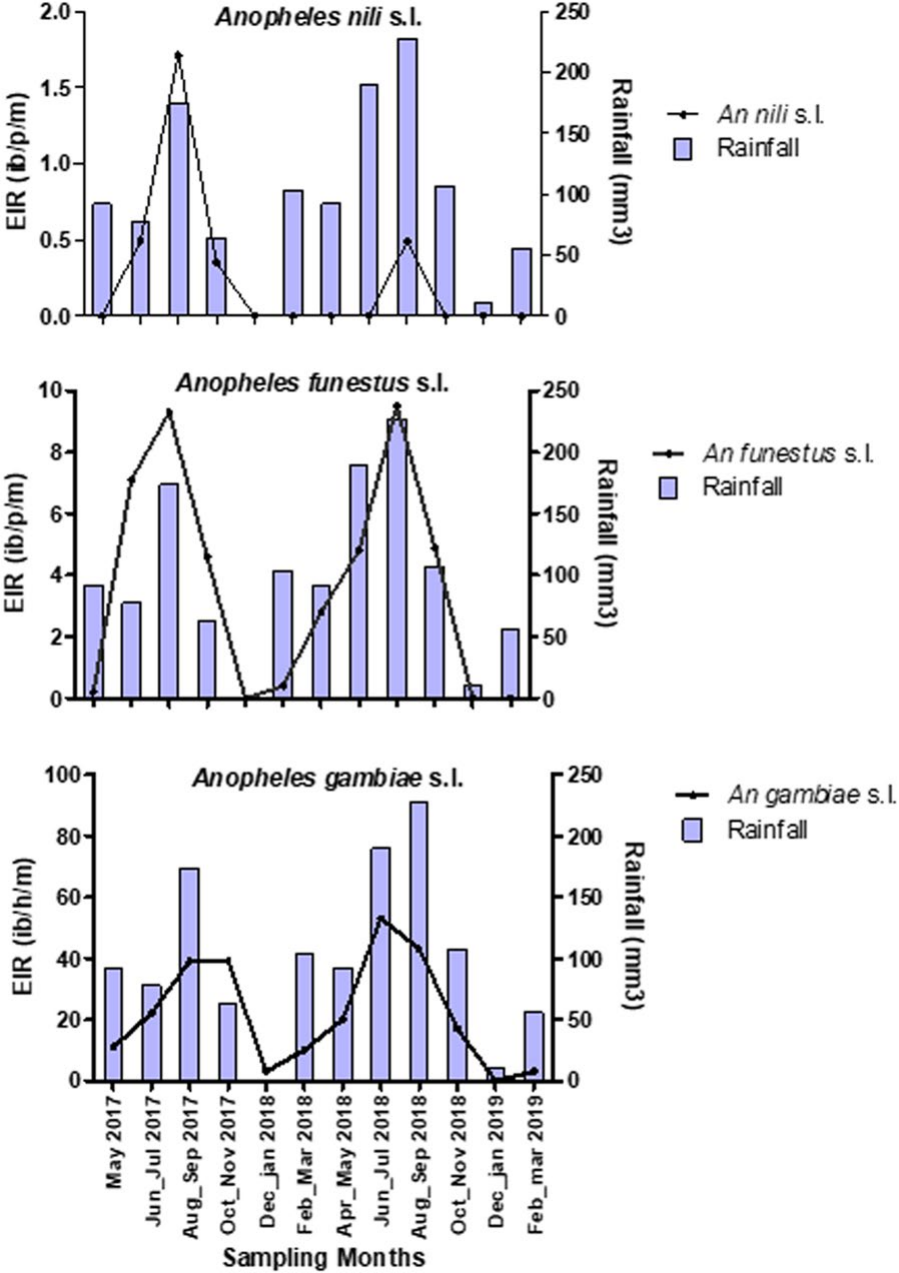
Monthly variation of entomological inoculation rate for Anopheles species from may 2017 to april 2019

In Gbêkê region, malaria transmission occurred all year long (Fig. 5), with variation in transmission intensities across the seasons (Table 2). From May 2017 to April 2019, the average annual EIR was estimated at 260.0 infective bite/per person/per year for *An. gambiae*; 43.5 ib/p/yr for *An. funestus* and 3.0 ib/p/yr for the *An. nili,* respectively. Monthly EIR was higher in the rainy season compared to the dry season, for both *An. gambiae* (30.6 [95% CI 27.3–33.9] *vs.* 3.9 [95% CI 2.4–5.4]) and *An. funestus* (5.4 [95% CI 4.7–6.1] *vs.* 0.1 [95% CI 0.0–0.2]; Table 2). Transmission intensity reached its peak in August–September, with an average of 46.0 ib/p/m for *An. gambiae*, 9.4 ib/p/m for *An. funestus* and 1.1 ib/p/m for *An. nili* (Fig. 5). Overall, *An. gambiae* was the major malaria vector responsible for 84.8% of total transmission, followed by *An. funestus*: 14.9% of transmission (Table 2). These vectors were responsible for malaria transmission in both the rainy season and the dry season. *Anopheles nili* (0.98%) also played an active role in the transmission of malaria parasites in rainy season, although its importance is far less than that of *An. gambiae* and *An. funestus*.

## Discussion

This study was conducted to characterize *Anopheles* vector distribution and malaria transmission dynamics, and vector biting behaviour in Gbêkê region, central Côte d’Ivoire. High species diversity grouped into six genera of mosquitoes was recorded in the study area. The diversity and abundance of mosquito fauna observed in this study might result from favorable environmental conditions for the developement of mosquito in the study area.[37].

The study of malaria transmission revealed that three common African *Anopheles* vector, *An. gambiae*, *An. funestus* and *An. nili*, are involved and sustain parasite transmission to local communities. *Anopheles gambiae* is the primary vector in the area, accounting for 84.8% infective bites. These data indicate high *Plasmodium* infection rates in *An. funestus*, affirming its role as the important vector in the location particulary in the village of Kouassi Atienkro, M’Mebo and Gare Kan. *Anopheles nili* were also found infected with malaria parasites, but it was present at a very low density. Infection levels recorded with *An gambiae* during this study were close to those previously recorded in the same region of Côte d’Ivoire [31, 38]. However, the involvement of *An. funestus* and *An. nili* in malaria transmission alongside *An. gambiae* in the area contrasted with the recent findings, where transmission was mainly sustained by *An. gambiae* [30, 31]. Indeed, these species responsible for all the *Plasmodium* ssp transmission recorded in this study have previously been incriminated in malaria transmission in Côte d’Ivoire [17, 19, 20, 39, 40].

This study demonstrated that the malaria vector species and abundance and malaria transmission intensity in the Gbêkê region varied significantly according to the season. *Anopheles gambiae* was present all year long; however, it was found at very low density during the dry season but became very abundant in the rainy season. Indeed, the larval habitats of this species are known to increase in number and productivity in rainy season but appear to diminish significantly during the dry season [41]. *Anopheles funestus* densities decreased also during the dry season. This is expected for *An. gambiae*, but is surprising for *An. funestus*. It has been described that *An. funestus* reaches its peak of abundance during the dry season in Savannah areas [42]. The larvae of this species is commonly found in large, permanent or semi-permanent bodies of fresh water such as swamps, large ponds and lake edge, preferentially with emergent vegetation on its margins [43]. In the study area, overall, the natural swamps and marshes are the most important potential breeding sites, and the extent of these habitats depends predominantly on the rains, explaining the low density of *An. funestus* during the dry season. Nevertheless, the high sporozoite rate recorded for *An funestus* in the dry season despite its low density suggested that this species strongly contributes to maintain malaria transmission during this season. *Anopheles nili* is also present in the area during the rainy season, although at a very low density. It was collected more particularly in certain villages (Kouassi Atienkro, M’Mebo and Gare Kan) close to several rivers and the water level of these rivers is kept high in rainy season. This reflects the presence of larval habitats favourable to the development of this species [20, 44]. But *An. nili* has greatly diminished in the year 2 of the collection of mosquitoes even though there was abundant rain. This is because excessive rainfall could also flush out breeding sites thus reduces the mosquito population [45, 46].

High risk of malaria transmission was recorded in Gbêkê region probably due to the presence of several vectors harbouring the *Plasmodium* parasite. These results estimated that unprotected individual living in Gbêkê region could receive an average of more than 321 infective bites per year from three major vector species (*An. gambiae*, *An. funestus* and *An. nili*) despite high coverage of LLINs. This high EIRs are consistent with previous work [31, 38], have also been reported from other regions of Côte d'Ivoire [17, 19]. Such levels of transmission recorded in the country, are relatively high when put in African context [47, 48]. The risk of being bitten by malaria vector mosquitoes was found to be up to ninefold higher during the rainy season compared to dry season. The increase in EIR in the rainy season could be explained by the increase in vectors densities and sporozoite rate during this season. Similar observations were reported elsewhere [42]. It could also be that environmental temperature plays a role as temperatures during the dry season are potentially above the optimum for malaria transmission [49, 50], contributing further to the observed seasonality.

Hourly mosquito captures showed that malaria vector populations began host searching at around 06:00 pm –07:00 pm, peaked at 00:00 am–03:00 am and then declined to negligible levels by 06:00 am–07:00 am. The biting time does not indicate a shift in host seeking towards dusk or dawn when people are unprotected by their bed nets**.** However, it was observed that *An. gambiae* seems more likely to feed outdoors than indoors that is in accordance with others results recorded in northern Côte d’Ivoire [17]. Endophagy is usually the expected dominant behaviour in *An. gambiae* [51–53]. It would appear that insecticide pressure from IRS and ITNs is selecting for mosquito vector populations which are increasingly outdoor feeding [54–56]. Some studies have shown that social patterns and human behavior (in terms of sleeping hours, outdoor activities and ITN use) may determine exposure to *Anopheles* mosquitoes and have an effect on transmission [57, 58]. Previous findings in the study area revealed that peoples spend a substantialamount of time outdoors [58] so there are potentially many opportunities for exposure when householders are not necessarily indoors and protected by LLINs. Indoor vector control measures alone (such as LLINs and IRS) could target a significant part of the vector population but are unable to stop transmission [59]. Hence, malaria control in high endemic areas needs to be strengthened with complementary tools to alleviate the burden of the disease. One limitation of the study was the use of qPCR which has been shown to overestimate the sporozoite rate [60].

This study has allowed a better understanding of malaria transmission dynamics and vector biting behaviour in Gbêkê region following the universal coverage of LLINs. The study highlights the risk factors of transmission that could negatively impact current interventions that target indoor control. Considering an aim of malaria elimination in Côte d’Ivoire and particulary in the Gbêkê region, it is increasingly urgent to research and develop novel vector control tools or complementary strategies particularly designed to suppress its very large malaria vector populations and the behaviour of vector populations.

## Conclusions

Malaria transmission in the Gbêkê area was mainly due to *An. gambiae*, while *An. funestus* group and *An. nili* complex played minor roles. This is the first report on the contribution of the *An. nili* as a secondary vector of malaria transmission in the area. The entomological indicators of malaria transmission were high despite the presence of standard LLINs. Additional vector control tools are urgently needed to complement current malaria control interventions.

## Data Availability

The datasets used and/or analysed during the current study are available At Intitut Pierre Richet/Institut national de santé Publique and will be made available on reasonable request.

## Abbreviations

CRT: Cluster randomized trial
HLC: Human landing catches
EIR: Entomological inoculation rate
SR: Sporozoite infection rate
LLINs: Long-lasting insecticide nets
IRS: Indoor residual spraying
NMCP: National malaria control programme
SET: Sreening plus eave tube
PCR: Polymerase chain reaction
